# Prediction of Speech Sounds Is Facilitated by a Functional Fronto-Temporal Network

**DOI:** 10.3389/fncir.2018.00043

**Published:** 2018-05-23

**Authors:** Lena K. L. Oestreich, Thomas J. Whitford, Marta I. Garrido

**Affiliations:** ^1^Queensland Brain Institute, The University of Queensland, Brisbane, QLD, Australia; ^2^Centre for Advanced Imaging, The University of Queensland, Brisbane, QLD, Australia; ^3^School of Psychology, University of New South Wales, Sydney, NSW, Australia; ^4^Australian Centre of Excellence for Integrative Brain Function, The University of Queensland, Brisbane, QLD, Australia; ^5^School of Mathematics and Physics, The University of Queensland, Brisbane, QLD, Australia

**Keywords:** predictive coding, electroencephalography (EEG), dynamic causal modeling (DCM), effective connectivity, structural connectivity

## Abstract

Predictive coding postulates that the brain continually predicts forthcoming sensory events based on past experiences in order to process sensory information and respond to unexpected events in a fast and efficient manner. Predictive coding models in the context of overt speech are believed to operate along auditory white matter pathways such as the arcuate fasciculus and the frontal aslant. The aim of this study was to investigate whether brain regions that are structurally connected via these white matter pathways are also effectively engaged when listening to externally-generated, temporally-predicable speech sounds. Using Electroencephalography (EEG) and Dynamic Causal Modeling (DCM) we investigated network models that are structurally connected via the arcuate fasciculus from primary auditory cortex to Wernicke’s and via Geschwind’s territory to Broca’s area. Connections between Broca’s and supplementary motor area, which are structurally connected by the frontal aslant, were also included. The results revealed that bilateral areas interconnected by indirect and direct pathways of the arcuate fasciculus, in addition to regions interconnected by the frontal aslant best explain the EEG responses to speech that is externally-generated but temporally predictable. These findings indicate that structurally connected brain regions involved in the production and processing of auditory stimuli are also effectively connected.

## Introduction

The ability to predict imminent sensations from past experiences such as hearing a familiar song, is crucial to efficiently process the abundance of sensory stimulation we experience at any moment. Moreover, it enables rapid detection of unexpected events and facilitates adaption to novel contingencies in our environment (Mumford, 1991, 1992). The predictive coding framework posits that in an effort to optimize sensory processing, the brain continuously generates models of the environment that are based on memories specific to a given context (Friston, 2005; Garrido et al., 2007). According to this theory, predictions are generated in higher cortical areas and communicated to lower sensory areas via backward (top-down) connections. The sensory areas then compare actual sensory input with the predicted sensation and its difference, i.e., mismatch or prediction error, is conveyed upstream via forward (bottom-up) connections (Rao and Ballard, 1999). This prediction error signal facilitates continuous updating of the internal predictive model.

The functional anatomy underlying auditory prediction is yet to be conclusively determined. One of the primary ways that humans produce sounds is by vocalizing (e.g., speaking). It is plausible that the neural architecture involved in producing and perceiving willed speech overlaps with the neural architecture involved in predicting sounds more generally (Gagnepain et al., 2012). The arcuate and aslant fasciculi are two white matter fiber bundles that are potentially involved in predictive coding in the context of willed speech. The arcuate fasciculus provides a direct connection between speech production (Broca’s) and speech perception (Wernicke’s) areas. In addition to direct, long segment fibers connecting Broca’s and Wernicke’s area, the arcuate fasciculus also has shorter, indirect connections consisting of an anterior pathway which connects Broca’s area to Geschwind’s territory, and a posterior pathway which connects Geschwind’s territory and Wernicke’s area (Catani et al., 2005). These long and short distance pathways of the arcuate fasciculus possess different functional roles: whereby the direct pathway is thought to be involved in phonological functions, the indirect pathways are associated with semantic functions (Catani and ffytche, 2005). Specifically, the posterior indirect pathway is thought to be involved in auditory comprehension and the anterior indirect pathway in the vocalization of semantic information (Catani et al., 2005). Evidence for a role of the arcuate fasciculus in predictive coding in the context of willed speech comes from studies with schizophrenia patients (Whitford et al., 2017), which showed that the structural integrity of the arcuate fasciculus is associated with predictive coding deficits, as quantified by the level of electrophysiological suppression to willed speech. The frontal aslant, which directly connects Broca’s area with the supplementary motor area (SMA; Catani et al., 2012) may also play a role in predictive coding in the context of speech production, as it is known to be involved in verbal fluency (Catani et al., 2013) and speech initiation (Fujii et al., 2016).

According to the “forward model” of speech production, the sensory consequences of self-generated speech are predicted through a copy of the motor command, which is sent via top-down projections from the motor cortex to the sensory system (Houde and Jordan, 1998). If the mechanisms involved in predictive coding of external, predictable sounds operate via similar neural pathways as those involved in predictive coding of willed speech, then the former may rely on the functional engagement of the arcuate fasciculus and the frontal aslant.

In this study, we formulated a set of dynamic causal models (DCMs) to investigate the functional underpinnings of auditory prediction of external, predictable speech sounds. These DCMs included brain regions interconnected via the arcuate fasciculus and the frontal aslant. It was hypothesized that models with both forward (bottom-up) and backward (top-down) connections, which convey sensory input and prediction, respectively, would perform better than models with forward (bottom-up) connections alone. Furthermore, we explored whether auditory prediction was better explained by alternative models that included or excluded the above mentioned regions along the arcuate fasciculus (Geschwind’s territory) and the frontal aslant (SMA).

## Materials and Methods

### Participants

Seventy-five healthy participants (38% males, aged 18–44 years, 95% right-handed) were recruited through the online recruitment systems SONA-1 and SONA-P at the University of New South Wales (UNSW), Australia. Participants were either monetarily reimbursed for their time or received course credit. One participant was excluded from the analyses due to a self-reported diagnosis of an Axis I disorder (American Psychiatric Association, 2000). Event-related potential (ERP) analyses and a detailed description of the demographic data have been reported previously elsewhere (Oestreich et al., 2015). All participants gave written informed consent in accordance with the Declaration of Helsinki. This study was approved by the UNSW Human Research Ethics Advisory Panel (Psychology) and the University of Queensland Research Ethics Committee.

### Procedure

Participants completed a number of questionnaires about their demographics, alcohol, nicotine, caffeine and recreational drug use, as well as history of Axis I disorders. Participants then underwent electroencephalographic (EEG) recordings while performing an experimental task in a quiet, dimly lit room. The experiment consisted of three conditions, namely the *Talk*, *Passive Listen* and *Cued Listen* conditions (Ford et al., 2007; Oestreich et al., 2015). Before the experiment, an instruction video was played, which demonstrated how to vocalize the syllable “ah” in a clear manner while maintaining the gaze on a fixation cross. Following the instruction video, participants were trained to vocalize the syllable “ah” with a duration of less than 300 ms and an intensity between 75 dB and 85 dB. During the *Talk* condition, participants vocalized a series of “ah”s in a desk-mounted microphone, every one to three seconds until 3 min had elapsed, producing between 75 and 125 “ahs.” In the *Cued Listen* condition, participants were instructed to listen to a recording of their own willed vocalizations whilst watching a video of the vocalization waveforms. Participants were therefore able to make exact temporal predictions about the onset of a speech sound. Lastly, during the *Passive Listen* condition, participants listened to their own willed vocalizations played back without a cue. During the *Passive Listen* condition, participants were therefore unable to make temporal predictions about the onset of the next speech sound.

Of the three conditions, the *Talk* condition is distinct from the other two in that it alone involves an overt motor action. As we were interested in the functional connectivity changes associated with auditory prediction *per se*, the *Talk* condition was removed from the analysis, described below, in order to avoid the complications associated with comparing motor-active and motor-passive conditions.

### Data Acquisition and Preprocessing

EEG was recorded with a 64-channel BioSemi ActiView system at a sampling rate of 2048 Hz, 18 dB/octave roll-off and 417 Hz bandwidth (3 dB). External electrodes were placed on the mastoids, the outer canthi of both eyes and below the left eye. EEG data were referenced to the average of the mastoid electrodes. Preprocessing was performed using SPM12 (Wellcome Trust Centre for Neuroimaging, London^1^) with MATLAB (MathWorks). Triggers were inserted at the onset of each “ah” and the EEG data were then segmented into 500 ms intervals with 100 ms pre- and 400 ms post-stimulus onset. Eye blinks and movements were corrected with a regression based algorithm using vertical and horizontal electrooculogram (VEOG, HEOG; Gratton et al., 1983). The low and high frequency components of the EEG signal were attenuated using a 0.5–30 Hz bandpass filter and trials containing artifacts exceeding ±50 μV were rejected. The remaining artifact free trials were averaged per condition for each participant in order to obtain event-related potentials (ERPs). ERPs were baseline corrected using the –100 to 0 ms pre-stimulus interval. The N1 component of each ERP was defined as the most negative peak between 50 ms and 150 ms after the onset of a speech sound. In order to investigate the effect of condition on N1 amplitude at electrode Cz, a paired-samples *t*-test with the within-subjects factor *condition* (Passive Listen/Cued Listen) was conducted.

### Dynamic Causal Modeling (DCM)

Dynamic causal modeling (DCM) relies on a generative spatiotemporal model for EEG responses evoked by experimental stimuli (Kiebel et al., 2008). It uses neural mass models (David and Friston, 2003) to infer source activity of dynamically interacting excitatory and inhibitory neuronal subpopulations (Jansen and Rit, 1995), and the connectivity established amongst different brain regions. DCM sources are interconnected via forward, backward and lateral connections (Felleman and Van Essen, 1991), and are arranged in a hierarchical manner (David et al., 2005; Kiebel et al., 2007). DCM is designed to test specific connectional hypotheses that are motivated by alternative theories (Garrido et al., 2008). Every connectivity model defines a network that attempts to predict (i.e., generate) the ERP signal. Differences in the ERPs to different experimental stimuli are modeled in terms of synaptic connectivity changes within and between cortical sources (Garrido et al., 2008). Several plausible cortical network connections are compared by estimating the probability of the data given a particular model within the space of models compared, using Bayesian Model Selection (BMS; Penny et al., 2004). BMS provides estimates of the posterior probability of the DCM parameters given the data, as well as the posterior probability of each model (Penny et al., 2004). The winning model is the model, which maximizes the fit to the data while simultaneously minimizing the complexity of the model.

The posterior probability of each model was computed over all participants using a random effects approach (RFX; Stephan et al., 2009). The conventional fixed effects approach for model comparison is limited by the assumption that all participants’ data are generated by the same model and is not very robust to outliers. The RFX approach used in the current study on the other hand, is able to quantify the probability that a specific model generated the data for any randomly chosen participant relative to other models. Moreover, RFX is robust to outliers (Stephan et al., 2009). We report the expected probability, that is, the probability that a particular model generated the data of a randomly chosen subject and the exceedance probability, which is the probability that one model is more likely than any other model, given the group data (Stephan et al., 2010). The main conclusions are based on inferences at the family level with a RFX exceedance probability of 0.95 on average (ranging from 0.85 to 1). In addition to RFX, we also report the Bayesian omnibus risk (BOR), which quantifies the risk incurred when performing Bayesian model selection, by directly measuring the probability that all model frequencies are equal (Rigoux et al., 2014). The BOR is bounded between 0 and 1, whereby a value close to 1 indicates that the models are indistinguishable, whereas a value close to 0 indicates that the models are well distinguishable from one another.

### Model Specification

**Figure 1:**
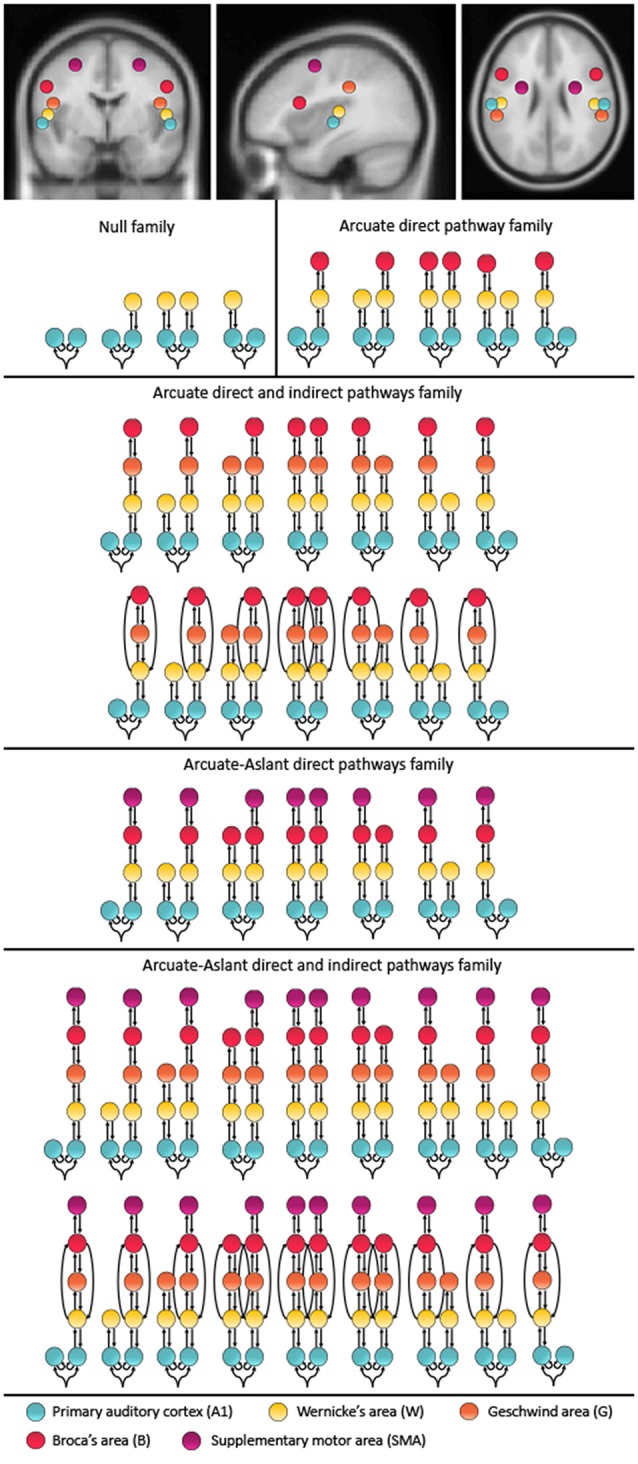
Mean locations for the dynamic causal modeling (DCM) nodes and model space. The montreal neurological institute (MNI) coordinates include: left A1 (−52, −19, 7), right A1 (50, −21, 7), left W (−57, −20, 1), right W (54, −19, 1), left G (−53, −32, 33), right G (51, −33, 34), left B (−48, 13, 17), right B (49, 12, 17), left supplementary motor area (SMA; −28, −2, 52), SMA (28, −1, 51). The 48 represented models were included twice, once with *forward* connections only and once with *forward and backward* connections. These 96 models were chosen to test different hypotheses about the functional anatomy of predictability to temporally cued speech. The models were combined into five families including a *Null family*, the *Arcuate direct pathway family*, the *Arcuate direct and indirect pathways family*, the *Arcuate-Aslant direct pathways family* and the *Arcuate-Aslant direct and indirect pathways family*.

The models compared in this study include up to 10 brain regions hierarchically organized in one to five levels. These alternative models were motivated by speech-related brain regions that are interconnected via the auditory white matter pathways of the arcuate fasciculus and the frontal aslant. Furthermore, these brain regions have previously been reported to be activated during auditory prediction tasks similar to the paradigm used in the present study. Specifically, a study using concurrent EEG and fMRI found the superior temporal gyrus (STG), which includes Wernicke’s area (W) and the primary auditory cortex (A1; Ford et al., 2016) to be activated, and a study using EEG with anatomical MRI reported activity in the STG, sensorimotor area and inferior frontal gyrus, which includes Broca’s area (B; Wang et al., 2014). Since the primary auditory cortex is essential for processing auditory information, the bilateral primary auditory cortices (A1) were defined as the cortical input nodes. The arcuate fasciculus consists of a direct pathway between Wernicke’s area (W) and Broca’s area (B) as well as two indirect pathways, namely the posterior pathway connecting W and the Geschwind’s territory (G), and the anterior pathway connecting G and B. To account for these direct and indirect connections of the arcuate fasciculus, we included models with and without G. Given the role of the frontal aslant in verbal fluency (Catani et al., 2013) and speech initiation (Fujii et al., 2016), models along the frontal aslant, which connects B with the SMA, were also included. The coordinates were chosen based on the mean Montreal Neurological Institute (MNI) coordinates for left A1 (−52, −19, 7), right A1 (50, −21, 7), left W (−57, −20, 1), right W (54, −19, 1), left G (−53, −32, 33), right G (51, −33, 34), left B (−48, 13, 17), right B (49, 12, 17), left SMA (−28, −2, 52) and right SMA (28, −1, 51; see Figure 1).

Since the effective connectivity associated with the prediction of external speech sounds has not been studied before, we considered a comprehensive model space including a total of 96 models comprising symmetric and non-symmetric hierarchical models, with forward (bottom-up) connections only and combined forward (bottom-up) and backward (top-down) connections, with and without indirect connections between W and B via G, as well as models with and without connections along the frontal aslant, which connects B to SMA (for a full description of the model space see Figure 1). All models allowed for changes of intrinsic connectivity at the level of A1 and were estimated and individually compared to each other using BMS. The 96 models were then partitioned into a number of different families.

We investigated whether the prediction of external, predictable sounds is driven by feedback loops, through both forward and backward connections, or by bottom-up inputs alone, via forward connections between brain regions along the arcuate fasciculus, and possibly also through the frontal aslant. Models with feedback loops would support the predictive coding framework whereby internal predictive models are constantly updated by prediction errors resulting from the mismatch between predicted and actual auditory sensations. To this end, a family consisting of all 48 models with forward connections (i.e., *Forward family*) only was compared to a family consisting of all 48 models with forward and backward connections (i.e., *Forward and Backward family*).

**Figure 2:**
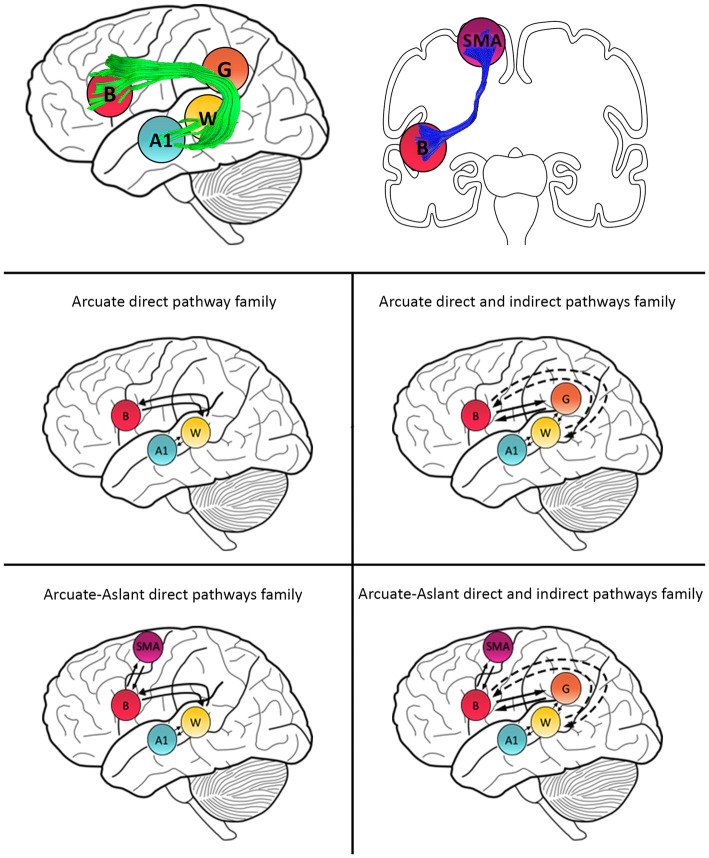
Schematic representation of family definitions and anatomical white matter pathways. Primary auditory cortex (A1), Wernicke’s area (W), Geschwind’s territory (G) and Broca’s area (B) are interconnected via the arcuate fasciculus (green). B and SMA are interconnected by the frontal aslant (blue).

Models were then grouped into families that included specific regions defined along auditory white matter tracts as follows: (1) the *Null family* consisted of eight models that included A1 only and models connecting A1 to W; (2) the *Arcuate direct pathway family* included 10 models, with connections between A1 and W as well as W and B; (3) the *Arcuate direct and indirect pathways family* consisted of 28 models including connections between A1 and W, W and G, G and B, as well as W and B; (4) the *Arcuate-Aslant direct pathways family* included 14 models with connections between A1 and W, W and B, as well as B and SMA; and (5) the *Arcuate-Aslant direct and indirect pathways family* comprising 18 models, including connections between A1 and W, W and G, G and B, W and B as well as B and SMA (see Figures 1, 2).

To follow up whether models with or without the frontal aslant (i.e., connections to SMA) better explained speech sound prediction, we first combined the *Arcuate direct pathway family* (10 models with connections linking A1, W and B directly; see Figures 1, 2) and the *Arcuate direct and indirect pathways family* (28 models linking A1, W, G and B) into one single family—the *Arcuate family*. We then compared this to the *Arcuate-Aslant family*, which resulted from combining the *Arcuate-Aslant direct pathways family* (14 models) and the *Arcuate-Aslant direct and indirect pathways families* (36 models) consisting of all the 50 models with connections to SMA (see Figures 1, 2).

Lastly, to investigate whether Geschwind’s territory is part of the circuit engaged in speech sound prediction, we compared families of models with and without connections to Geschwind’s territory. To this end, we combined all models excluding Geschwind into one family—*no Geschwind family—*by grouping the *Arcuate direct* *pathway family* (10 models) and the *Arcuate-Aslant direct pathways family* (14 models; see Figures 1, 2). We compared then the *no Geschwind family* to the *Geschwind family*, which included a combination of the *Arcuate direct and indirect pathways family* (28 models) and the *Arcuate-Aslant direct and indirect pathways family*, that is, all the models that included Geschwind’s territory (36 models). Each of the 96 models was fitted to each individual participant’s mean response for the contrast between the *Passive Listen* and *Cued Listen* conditions, whereby the *Passive Listen* condition was used as the baseline condition.

## Results

### Scalp Analysis

**Figure 3:**
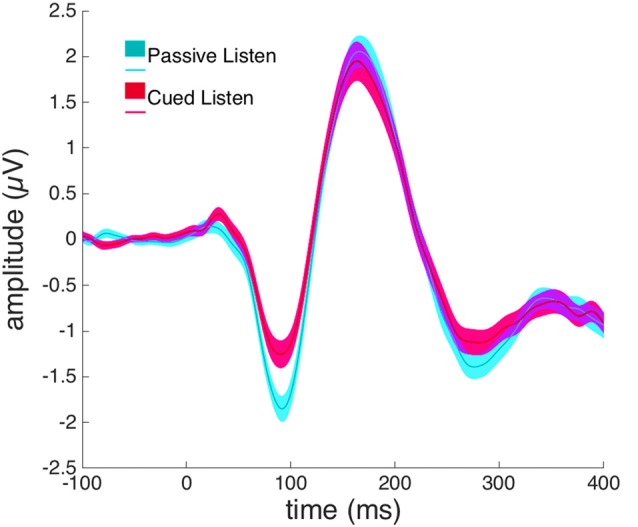
Event-related potentials (ERPs) from electrode Cz in response to willed vocalization in the Cued Listen (magenta) and Passive Listen (cyan) conditions.

A paired-samples *t*-test revealed a significant difference between the *Passive Listen* and *Cued Listen* conditions on the N1-amplitude at electrode Cz (*t*_(72)_ = 2.460, *p* = 0.016, *Cohen’s d* = 0.288; see Figure 3).

### DCM Analyses

**Figure 4:**
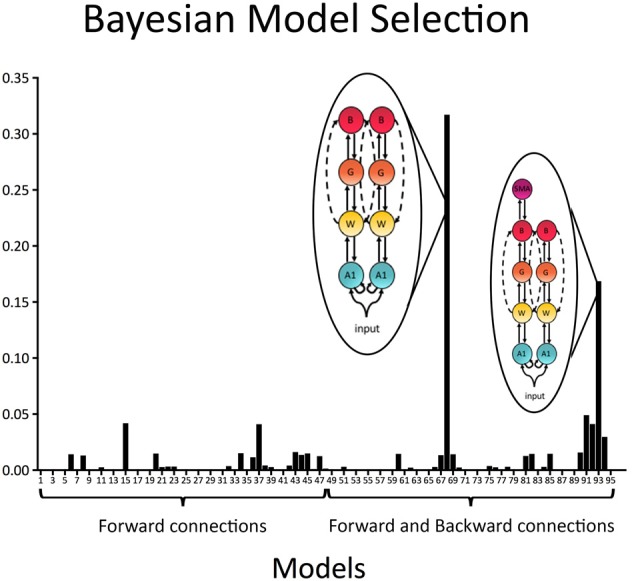
Model exceedance probability for attenuation of predictable speech. Bayesian model selection (random effects) over the whole model space indicated speech sound prediction was best explained by a model with recurrent (i.e., forward and backward) connections between bilateral primary auditory cortex (A1), Wernicke’s area (W), Geschwind’s territory (G) and Broca’s area (B), as well as direct bilateral connections between W and B. This model was followed by a model, which was in all equal to the winning model except that it included a connection from B to SMA in the left hemisphere.

In a first step all 96 models with forward (bottom-up) connections only as well as forward (bottom-up) and backward (top-down) connections were individually compared to each other. Results indicated that the best model included recurrent connections linking A1, W, G and B, as well as direct connection between W and B in both the left and right hemispheres (exceedance probability = 0.32; BOR < 0.01; see Figure 4). The second-best model, which was also relatively probable, was equal to the winning model except that it did include connections to SMA via the aslant in the left hemisphere (exceedance probability = 0.17; see Figure 4).

When comparing a family with modulations of forward (bottom-up) connections only (i.e., *Forward family*) to a family of both forward (bottom-up) and backward (top-down) connections (i.e., *Forward and Backward* *family*), we found that the family consisting of a combination of *Forward and Backward* connections (expected probability = 0.56, exceedance probability = 0.85) better explained speech sound prediction than the families including *Forward* connections only.

**Figure 5:**
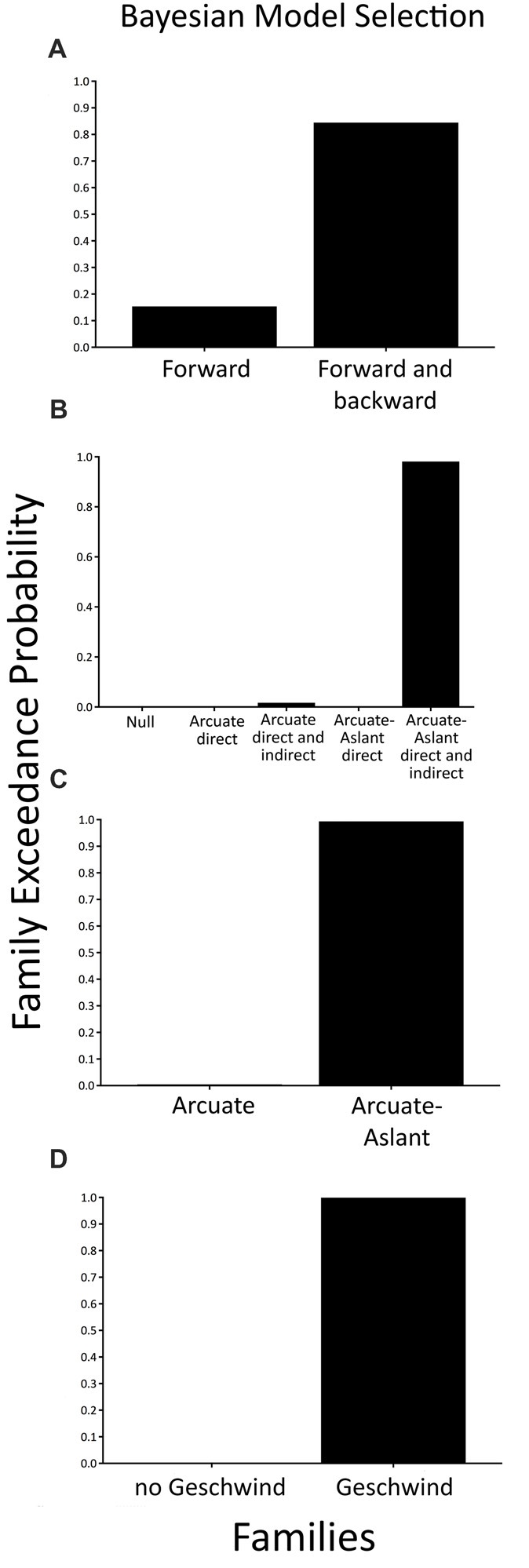
Family-level inference for attenuation of predictable speech sounds—exceedance probabilities for the family comparisons. **(A)** Comparison of the *Forward family* (48 models) to the *Forward and Backward family* (48 models). **(B)** Comparison of five families including a *Null family* (eight models), the *Arcuate direct pathway family* (10 models), the *Arcuate direct and indirect pathways family* (28 models), the *Arcuate-Aslant direct pathways family* (14 models), and the *Arcuate-Aslant direct and indirect pathways family* (18 models). **(C)** Comparison of the *Arcuate family* (38 models) and the *Arcuate-Aslant family* (50 models). **(D)** Comparison of *no Geschwind family* (24 models) to *Geschwind family* (64 models).

To test specific hypotheses as to which brain regions that are interconnected by the arcuate fasciculus and the frontal aslant were engaged during the prediction of external, temporally-predictable speech sounds, five families of models were compared as described in the methods section (see Figures 1, 2). BMS of these families indicated that the *Arcuate-Aslant direct and indirect pathways family* was the winning family (expected probability = 0.54, exceedance probability = 0.98; see Figure 5).

When comparing families with the arcuate fasciculus alone (i.e., *Arcuate family*) to families including both the arcuate fasciculus and the frontal aslant (i.e., *Arcuate-Aslant family*), BMS revealed that the winning, *Arcuate-Aslant* *family* was much more likely than the *Arcuate* *family* (expected probability = 0.60, exceedance probability = 0.95; see Figure 5).

Lastly, we investigated families of models with and without Geschwind’s territory, which enquired as to whether Geschwind’s territory plays a role in the functional circuit engaged in speech sound prediction (*Geschwind family* vs. *no Geschwind family*). Results indicated that the family of models including connections to Geschwind’s territory outperformed the family of models without Geschwind’s territory (expected probability = 0.88, exceedance probability = 1; see Figure 5).

## Discussion

This study investigated the functional anatomy underlying temporally predictable speech sounds using DCM. Model comparison revealed that modulations with both forward (bottom-up) and backward (top-down) connections better explained speech sound prediction than forward (bottom-up) connections alone. Connectivity models linking primary auditory cortex, Wernicke’s area, Geschwind’s territory and Broca’s area via the arcuate fasciculus and the SMA, through the frontal aslant tract, outperformed models without connections to the SMA and Geschwind’s territory. These findings indicate that the circuitry underlying the prediction of temporally predictable, external sounds may involve brain regions involved in the prediction of willed speech, and may include both, the arcuate fasciculus and the frontal aslant.

The finding that a combination of forward (bottom-up) and backward (top-down) connections better explained the results than forward (bottom-up) connections alone is in line with the predictive coding account, whereby a prediction is conveyed through backward (top-down) connections. Forward connections can be conceptualized as bottom-up processes (Friston, 2005; Chen et al., 2009), which convey environmental sensory information from the primary auditory cortex to higher cortical levels. On the contrary, backward connections represent top-down (Chen et al., 2009), predictive processes based on self-monitoring or past experiences. In this study, we used a *Passive Listen* condition whereby participants were passively listening to a series of previously recorded vocalizations. We used this condition as a baseline and compared it to a *Cued Listen* condition, whereby participants were cued to the exact onset of each speech sound. Therefore, participants were able to make temporal predictions about the exact onset of each speech sound, which may have been transmitted through top-down, or backward connections along the arcuate fasciculus. On the contrary, during the *Passive Listen* condition, participants were unable to make temporal predictions about to the onset of the external sounds.

In line with these findings, Hickok (2013) proposed that the rapidity of production and comprehension of human dialog is only possible through predictive mechanisms, whereby listeners covertly imitate speakers based on their own internal representation of an utterance via top-down connections. This enables the listener to predict what the speaker is likely to say next. This theory is supported by the findings from this study whereby changes in effective connectivity from the *Passive Listen* condition to the *Cued Listen* condition are best explained by a feedback loop comprising conjoint forward (bottom-up) and backward (top-down) connections.

Another key finding of this study is that a family of models including brain areas and connections along the arcuate fasciculus (linking primary auditory cortex to Wernicke’s area and Broca’s area directly, and indirectly via Geschwind’s territory) and the frontal aslant (connecting Broca’s area directly to the SMA) best explained the prediction of temporally predictable, externally-presented speech sounds. When comparing all individual models, the winning model included connections along the arcuate fasciculus bilaterally. The second most probable model included additional connections to the frontal aslant in the left hemisphere, but only connections along the arcuate fasciculus in the right hemisphere. In order to determine whether the frontal aslant adds to the functional anatomy of speech sound prediction or whether connections along the arcuate fasciculus alone are sufficient, we compared families of all models with and without connections along the frontal aslant (while keeping the arcuate fasciculus pathways intact). The findings indicated that models with connections along the arcuate fasciculus and the frontal aslant better explained speech sound prediction than models including the arcuate fasciculus only. It may appear surprising that the family of models including the frontal aslant best explained sound prediction as the frontal aslant is thought to transmit the motor act of speech production and the *Cued Listen* condition did not involve a motor act. A possible explanation for the involvement of connections to the SMA and therefore the frontal aslant is a proposal put forward by Jackson (1958): since internal models of auditory predictions work reliably during processes of sensory motor control, the same internal models of auditory predictions, developed later in evolution, might also be utilized during higher cognitive processes such as thought or inner speech, which can be seen as the most complex motor act without actions. In the context of the present study, while participants were not actively generating the vocalization, watching the waveforms of the speech sounds might lead them to internally simulate the next vocalization, which might explain the activation of the SMA without a motor act. However, we acknowledge that this explanation is highly speculative, and should be treated with caution until supporting evidence is provided.

The arcuate fasciculus consists of long distance fibers which connect Broca’s and Wernicke’s area as well as short distance fibers which connect Broca’s and Geschwind’s territory via an anterior pathway, and Geschwind’s territory and Wernicke’s area via a posterior pathway (Catani et al., 2005). The results of the present study indicate that models including long distance connections in addition to short distance connections, via Geschwind’s territory, better explained sound prediction than models including long distance connections only. The direct, long distance pathway is thought to be involved in phonological repetitions (Catani and ffytche, 2005) and therefore represents a plausible connection to be utilized during this experimental tasks, whereby the same sound (i.e., a speech fragment) was played repetitively. The indirect, short distance pathways of the arcuate fasciculus are thought to be involved in semantic functions (Catani and ffytche, 2005). The engagement of these connections during the prediction of externally-presented speech sounds might be explained by the nature of the speech sounds used in the present study. Since phonemes are the building blocks of language which are used to distinguish one word from another, it is possible that participants assigned semantic meaning to these sounds, which would likely not occur if the sounds were simple tones.

The involvement of brain areas interconnected via the arcuate fasciculus during the prediction of externally-presented speech sounds is in line with findings from studies of speech sound prediction in schizophrenia. There is substantial evidence that patients with schizophrenia possess disrupted predictive coding mechanisms to self-generated speech (Ford et al., 2001, 2007; Ford and Mathalon, 2004), button-press elicited sounds (Whitford et al., 2011; Ford et al., 2014), and temporally cued sounds (Ford et al., 2007). Individuals at high-risk for developing a psychotic disorder show auditory predictive coding that is intermediate between healthy participants and patients with schizophrenia (Perez et al., 2012) and healthy individuals with psychotic-like experiences show reduced auditory predictive coding mechanisms compared to healthy individuals without psychotic-like experiences (Oestreich et al., 2015, 2016).

The mechanisms underlying these speech sound prediction deficits in schizophrenia and psychosis are still unclear. However, several studies have reported changes to the white matter structure, and specifically to the myelin sheath, of the axons constituting the arcuate fasciculus in patients with schizophrenia (Kubicki et al., 2005; Uranova et al., 2007). This is important insofar as it indicates that connectivity along the arcuate fasciculus during speech sound prediction should be delayed due to a loss of conduction velocity induced by demyelination. Support for this contention comes from a study by Whitford et al. (2011), which reported that auditory prediction abnormalities typically exhibited by patients with schizophrenia could be completely eliminated by imposing a 50 ms delay between a self-generated button press and the delivery of a sound. This was interpreted to indicate that the predictions of sensory consequences resulting from the motor command, travelling along the arcuate fasciculus during auditory prediction, were delayed by 50 ms in the group of schizophrenia patients. Furthermore, the study reported that the degree to which auditory prediction improved as a result of the delay between button press and tone delivery was linearly correlated with white matter abnormalities in the arcuate fasciculus. Furthermore, a recent study reported that predictive coding mechanisms were also disrupted in early illness schizophrenia and clinical high-risk for psychosis individuals and that the level of predictive coding abnormalities was linearly related to the microstructure of the arcuate fasciculus (Whitford et al., 2017). The findings from the present study add further support for the role of the arcuate fasciculus during auditory predictions—in this case, in the prediction of temporally predicable, but externally-generated sounds—by showing that the brain regions that are structurally interconnected by the arcuate fasciculus are also effectively connected.

DCM presents some limitations, most notably, the number of alternative models likely to explain a dataset can be very large and as a consequence, the best model might be missed if the model space is not comprehensive enough (Lohmann et al., 2012). While this is true indeed for any modeling approach that performs exhaustive searches, the objective of DCM is to perform comparisons on theoretically motivated mechanistic accounts for a given brain process. The output of DCM is the computation of an estimate for the relative evidence of different models as well as estimates about model features (i.e., connectivity parameters), rather than the specification of the single best model, which would generally have a rather small relative evidence in a large model space (Friston et al., 2013). However, to date, DCM is the only approach that integrates biophysical models of dynamic neural networks into statistical tools to investigate neuroscientific questions.

In this article we have inverted a large number of models that provided alternative mechanistic explanation for our data. Friston et al. (2016) recently introduced a new method for the analysis of group level DCM studies, which enables model selection while eschewing the need to invert all models explicitly. This approach uses parametric empirical Bayes (PEB) and Bayesian Model Reduction (BMR) to compute the posterior densities over all model parameters, under new prior densities without inverting the model again. Friston et al. (2016) demonstrated that PEB may improve the accuracy of the parameter estimates. We suggest that the use of PEB as an alternative analysis approach and a replication of this study with alternative methods to infer functional connectivity from multichannel neural EEG signals, such as phase synchronization analyses (Junfeng et al., 2012) represent fruitful avenues for future research.

In summary, the present study showed that auditory prediction to externally generated speech sounds involve brain regions such as Wernicke’s area, Broca’s area and Geschwind’s territory, interconnected through the arcuate fasciculus via both short- and long-distance fibers, as well as the SMA, which is linked to Broca’s area via the frontal aslant. Critically, we found that the prediction of externally-generated speech sounds engaged feedback loops with conjoint forward (bottom-up) and backward (top-down) connections. This result is consistent with a predictive coding framework, in which predictions are generated in higher cortical areas and communicated to lower sensory areas via backward, or top-down connections. These results also suggest that passively listening to temporally-predictable speech sounds may lead to the production of inner speech and may engage predictions such as those believed to be involved in the production of overt speech.

## Author Contributions

LO designed the study, collected the data, undertook the literature review, performed the analyses and wrote the first draft of the manuscript. TW obtained funding and designed the study. MG helped with the data analysis and interpretation of results. All authors contributed to and have approved the final manuscript.

## Conflict of Interest Statement

The authors declare that the research was conducted in the absence of any commercial or financial relationships that could be construed as a potential conflict of interest.

## Footnotes

^1^http://www.fil.ion.ucl.ac.uk/spm/
